# Young adult Latino testicular cancer survivors: a pilot study of Goal-focused Emotion regulation Therapy (GET)

**DOI:** 10.1007/s00520-024-08960-y

**Published:** 2024-10-30

**Authors:** Michael A. Hoyt, Belinda Campos, Jose G. Lechuga, Michelle A. Fortier, Karen Llave, Marcie Haydon, Michael Daneshvar, Christian J. Nelson, Baolin Wu

**Affiliations:** 1 Department of Population Health & Disease Prevention, Joe C. Wen School of Population & Public Health, University of California, 856 Health Sciences Drive, Irvine, CA 92697-3957 USA; 2 Chao Family Comprehensive Cancer Center, University of California, Irvine, USA; 3 Institute for Interdisciplinary Salivary Bioscience Research, University of California, Irvine, USA; 4 Department of Chicano/Latino Studies, University of California, Irvine, USA; 5 Sue & Bill Gross School of Nursing, University of California, Irvine, USA; 6 Center On Stress & Health, University of California Irvine, Irvine, USA; 7 Department of Urology, University of California, Irvine, USA; 8 Department of Psychiatry and Behavioral Science, Memorial Sloan Kettering Cancer Center, New York, NY USA; 9 Department of Epidemiology and Biostatistics, University of California, Irvine, USA

**Keywords:** Latino, Hispanic, Young adult, Cancer, Testicular, Intervention, Simpatía, Acculturative stress

## Abstract

**Purpose:**

Young adult Latino testicular cancer survivors experience adverse impacts after treatment. We developed Goal-focused Emotion regulation Therapy (GET) to improve distress symptoms, goal navigation skills, and emotion regulation. This open pilot trial extended GET to Latino young adult survivors of testicular cancer and assessed feasibility and tolerability as well as changes in anxiety and depressive symptoms. Secondary outcomes included goal navigation, emotion regulation, and components of hope-related goal processes (i.e., agency and pathway mapping). To assess the extent to which GET is culturally congruent or in need of adaptation, the influence of simpatía and acculturative stress were also examined.

**Methods:**

Thirty-five eligible young adult (age 18–39) survivors treated with chemotherapy were enrolled and assessed at baseline. Study acceptability, tolerability, and therapeutic alliance were examined. Preliminary efficacy was evaluated for changes in anxiety and depressive symptoms as well as psychological processes (goal navigation, agency, goal pathway skill, and emotion regulation) from baseline to immediate post- and 3-month post-intervention.

**Results:**

Among the 35 men assessed at baseline, 54% initiated intervention sessions. Among these, 94.7% completed all study procedures. Helpfulness ratings of intervention components and therapeutic alliance scores were strong. Repeated measures ANOVA revealed significant reductions in anxiety and depressive symptoms from pre- to post-intervention with sustained change at the 3-month follow-up. Favorable patterns of change were also observed in GET-related psychological processes. Simpatía was associated with less depressive symptoms at post-intervention, but not change in anxiety. Acculturative stress was associated with increased anxiety and depressive symptoms over time.

**Conclusion:**

GET is a feasible and acceptable intervention for reducing adverse outcomes after testicular cancer for young adult Latino men. Results should be considered preliminary but suggest meaningful changes in emotional and psychological outcomes.

## Introduction

Young adult (age 18 to 39) Latino men face unique challenges after cancer and experience higher levels of psychological distress and lower quality of life compared to non-Hispanic White (NHW) and Latina cancer survivors [1]. Despite these challenges, they are underserved in supportive cancer care and underrepresented in survivorship research, with Spanish-language monolinguals experiencing particularly limited access to services in Spanish [2]. This disparity highlights the urgent need to address the symptom burden and quality of life among young adult Latino men, considering both cultural and systemic factors. This urgency is further heightened because Latino men report worse cancer-related morbidities, including reduced sexual and physical functioning and worse mental health outcomes, compared to NHWs [2–16]. Despite this, Latino men engage less with supportive cancer care services compared to other patient groups [17, 18]. Altogether, there is a clear need for tailored evidence-based behavioral interventions, comprehensive survivorship care, and promotion of skills to manage ongoing cancer-related demands and the pursuit of important life goals [19].

Testicular cancer disproportionately impacts young adult men. New cases of testicular cancer for Latino men have increased by nearly 60%, compared to the only 1% annual increases observed in NHWs; this positions Latino men with the fastest-growing incidence [20]. The long-term adverse impacts of testicular cancer are more severe and persistent in those receiving chemotherapy and include physical symptoms (e.g., infertility, hearing loss), secondary malignancies, and chronic conditions of the endocrine, cardiopulmonary, and urogenital systems [21–29]. In addition, surgical procedures can increase the risk of secondary or permanent complications (e.g., abdominal scarring, hernia) [30–33]. The associated psychosocial impact is also substantial. A systematic review of studies of anxiety, depression, fear of recurrence, and distress among testicular cancer survivors found a higher prevalence in survivors than in the general population [34]. The prevalence of moderate to high anxiety ranges from 17 to 41% across studies, and clinically significant distress is as high as 5–20% [34–38]. About two-thirds of testicular cancer survivors report unmet survivorship needs [39–41] most commonly relating to supportive care, survivorship information, managing distress, fertility, relationships, self-image, and occupational problems [39, 40, 42–44]. The coping burden can be substantial, and these physical and psychosocial impacts can alter adult self-image [45] and delay, obstruct, or prevent engagement in goal pursuits.

Given that young adulthood is a critical time for goal attainment and self-concept development, cancer diagnosis during this period can significantly disrupt life goals and trajectories [46]. Moreover, re-entry to post-cancer life can be a critical point in the survivorship trajectory, and intervention at this time is well positioned to confer a longer-term impact. In studies of Latino male cancer survivors, positive emotion regulation, enhancement of personal agency, maintenance of self-efficacy, and balancing life goals with life demands have been identified as important and valued coping pathways [44]. These elements are especially useful to men in late adolescence and early adulthood, a life stage critical to the development of autonomy and self-concept. Thus, the focus on self-regulation through goal navigation capacity and emotion regulation as developmentally matched and possibly culturally congruent intervention targets is appropriate for this population [47–49].

Goal-focused Emotion regulation Therapy (GET) is a behavioral intervention developed to enhance self-regulation through improved goal navigation skills, improved sense of agency and purpose, and better ability to regulate emotional responses after cancer [50]. It is the only known intervention designed specifically to meet the needs of young adults after testicular cancer. GET focuses on identifying value-derived goals and learning skills to navigate a process of sustained movement toward them, including goal refinement, generating pathways toward goal fulfillment, and managing blocked or challenged strivings. The intervention is designed to foster agentic thinking and includes training in goal-related cognitive restructuring and emotion-regulating coping skills.

Preliminary effects of GET were tested in a randomized-controlled pilot trial of young adults within 2 years of completing chemotherapy for testicular cancer [46]. Relative to a supportive listening control, participants receiving GET had greater reductions in depressive (*d* = 0.45, *p* < 0.05) and anxiety (*d* = 0.29, *p* < 0.05) symptoms at post-intervention and 3 months later. Additionally, GET significantly increased goal navigation capacity and emotion regulation skills. Although formative work and feasibility research of GET included diverse samples [49, 51], GET was not developed with a strong cultural lens. Given the rapid increase in testicular cancer incidence among young adult Latinos [20], interventions like GET require consideration of cultural congruence and exploration of opportunities for optimization.

While the population of Latino testicular cancer survivors is growing, it is necessary to consider that cultural factors are likely to be relevant to their experiences of cancer-related psychosocial distress [e.g., 2–4]. There is thus a need to examine GET to understand whether it is culturally congruent for Latino testicular cancer survivors or needs cultural adaptation to be maximally effective. There is reason to expect that the cultural context of Latino testicular cancer survivors may influence the effectiveness of interventions like GET given that cultural values may impact how individuals respond to interventions [52]. In the context of Latino testicular cancer survivors, simpatía, which socializes an emphasis on positive emotions and avoiding conflict and social discomfort, and acculturative stress, resulting from experiences of discrimination and acculturation challenges, are both likely to be relevant. Both have the potential to evoke distinct patterns of emotion regulation behavior or other self-regulatory responses to stressful external or internal events.

Simpatía is a cultural value emphasizing experiencing and expressing positive emotions in social situations, a preference for interpersonally warm exchanges while simultaneously avoiding conflict and/or overt negativity [53]. Simpatía encourages a distinct pattern of emotion regulation and so carries the significant potential to shape the impact of GET. Research on Latino values and cancer highlights that cultural factors are seldom fully protective or exclusively deleterious [2]. For instance, simpatía can reduce conflict with health providers but can also thwart active engagement in cancer care [53, 54]. The GET intervention is designed to encourage self-regulation by enhancing the active pursuit of goals and expressing and managing difficult emotions. So how GET may or may not be congruent with simpatía-related patterns of emotion management (e.g., avoidance of negative emotions, preference for positive emotions, and harmony) will need to be understood.

The vulnerabilities associated with cancer diagnosis and survivorship in young adulthood may be exacerbated by the additional stressors of being a member of a marginalized ethnic group (e.g., acculturation processes). These stressors can include the pressure to conform to the norms of the dominant culture. For some, this involves aversive and health-harming experiences such as discrimination, rejection, or feelings of isolation or embarrassment [55]. Acculturative stress is the specific psychological and social stress experienced in reaction to such aversive experiences [55]. For young adult Latino men, acculturative stress has been shown to be associated with depression and anxiety by way of lower employment and utility of emotion regulation skills [47].

Research and clinical focus on young adult Latino men after cancer, including testicular cancer, is substantially lacking. The current pilot trial aimed to evaluate the acceptability, tolerability, and preliminary impact of GET among young adult Latino survivors of testicular cancer. Additionally, it seeks to explore the influence of cultural processes—simpatía and acculturative stress—on the impact of GET.

## Methods

### Trial design

This was a single-group, repeated measures open pilot trial approved by the institutional review boards at the University of California, Irvine and the California Health and Human Services Agency's Committee for the Protection of Human Subjects.

### Participants

Latino young adults with a testicular cancer diagnosis treated by chemotherapy were identified via the California Cancer Registry. Potential participants were recruited via informational letter and/or telephone call. Individuals were screened by a research assistant for eligibility. Eligible patients were between the ages of 18 and 39 years, had a confirmed diagnosis of testicular cancer (any stage), completed chemotherapy within 2 years prior, self-identified as Hispanic and/or Latino, and had English or Spanish fluency. Notably, the 2-year period after chemotherapy typically entails intensive surveillance because of the heightened risk of recurrence in this period [21, 56], which can be physically and psychologically taxing. Participants were also screened to exhibit sub-optimal self-regulation as evidenced by a score of 1.8 or below on the Goal Navigation Scale (see 35 for description of clinically meaningful thresholds) or a score of 4 or greater on the Distress Thermometer (DT) [57]. The Goal Navigation Scale of the Cancer Assessment for Young Adults (CAYA) has been designed and validated for young adult men with testicular cancer. It measures goal navigation skills, while the DT is a single-item visual analog screening tool for psychological distress with a 0 to 10 range in which a score of 4 or greater signals significant distress levels.

Exclusions included a lifetime history of severe mental illness (i.e., schizoaffective disorder, schizophrenia, psychosis), active suicidality, or impaired comprehension (e.g., dementia).

Participants were enrolled between May 2021 and May 2023.

### Procedures

Following written informed consent procedures, participants completed questionnaires via a secure HIPAA-compliant online platform and were then scheduled for intervention. All intervention sessions were delivered by trained mental health counselors with a minimum of master’s-level training who were bilingual in English and Spanish.

The six GET sessions were delivered over 8 weeks via video call, and participants were given at-home exercises via a participant workbook to be completed between sessions. Each of the six sessions was 60 min in length. The first four sessions were scheduled weekly, and the final two sessions were separated by 2 weeks to provide time for skill application. Intervention delivery was in strict accordance with the GET intervention manual, which has been described elsewhere in detail (see [50]). Briefly, session topics include a review of cancer-related experiences and influences on goal pursuits, psychoeducation regarding emotions, skills, and values (session 1), values clarifications and emotional awareness (session 2), achievability of goals, cognitive skills training (sessions 3), goal pathway mapping, navigating blocked goals and re-directing energy (sessions 4), goal motivation and agentic actions, self-care behavior (session 5), and goal pursuits moving forward (session 6).

A treatment integrity coding system was developed to assess the degree to which study interventionists adhered to the treatment protocol. Two independent raters evaluated audio recordings of each session. Across sessions, average fidelity scores ranged from 80 to 98%.

Prior to the delivery of GET in Spanish, all intervention materials were translated from English to Spanish by a certified translator. After initial translation, materials were then reviewed with bilingual (Spanish/English) young adults to identify phrasing that could be better communicated with conversational Spanish. Finally, GET was delivered in Spanish to pilot participants to examine acceptability prior to study recruitment.

Participants repeated questionnaires after the last intervention session and again 3 months later. Participants were given $50 at each data collection point.

### Measures

#### Acceptability and tolerability

Acceptability was indicated by the percentage of eligible men who consented to participation. To further quantify acceptability, participants were asked to rate the helpfulness of the intervention skills, number and length of sessions, homework assignments, and therapist interactions on a scale from 0 (did not help at all) to 5 (extremely helpful). They also rated the likelihood they would recommend this intervention to a friend with testicular cancer. Responses ranged from 0 (definitely not) to 5 (definitely). Finally, tolerability is reported as the percentage of men who completed study procedures.

#### Therapeutic alliance

Participants completed the Working Alliance Inventory-Short Form (WAI-SF) [58] at the immediate post-intervention assessment, which assesses the perceived strength of the treatment alliance. The WAI-SF includes 12 items (e.g., “I feel that my interventionist appreciates me”) on a response scale ranging from 1 (never) to 7 (always). Cronbach’s alpha was 0.83.

#### Outcome measures

The primary outcomes were anxiety and depressive symptoms as measured by the Hospital Anxiety and Depression Scale (HADS) [59]. The HADS is a 14-item questionnaire, with 7 items assigned to each of the HADS-Anxiety (HADS-A) and HADS-Depression (HADS-D) subscales. Each item is rated on a 4-point response scale (0 to 3). Subscale scores are categorized to indicate the level of anxiety or depression experienced where scores of less than 8 are categorized as normal, scores of 8–10 as borderline, and scores of 11–21 as clinically notable. Cronbach’s alpha ranged from 0.88 to 0.89 for HADS-A and from 0.78 to 0.82 for HADS-D.

#### Psychological processes

Secondary outcomes included self-report measures reflecting core GET processes, including goal navigation, emotion regulation, and components of hope (i.e., agency and pathway mapping).

#### Goal navigation processes

Goal navigation capacity and goal blockage were measured by the Cancer Assessment for Young Adults (CAYA) [35]. Goal navigation capacity includes elements of goal setting, goal clarification, adjustment, and initiation. The scale is composed of five items (e.g., “I am able to identify goals in my life,” “I know what steps to take to make progress toward my goals,” and “I am able to redirect my energy when I feel my life isn't going in the right direction”). Goal blockage assesses the degree to which one perceives their life goals are blocked or unobtainable because of cancer. The scale is composed of five items (e.g., “Cancer has made some goals unattainable,” “My goals are off-track because of my health”). Participants indicate how often each item is true for them over the past 7 days on a 3-point response scale ranging from 0 (*none of the time*) to 2 (*much or most of the time*). Cronbach’s alpha ranged from 0.71 to 0.88 for goal navigation capacity and from 0.73 to 0.81 for goal blockage.

#### Emotion regulation processes

Two emotion regulation processes, cognitive reappraisal and expressive suppression, were measured by the Emotion Regulation Questionnaire (ERQ). The ERQ is a widely used 10-item scale designed to measure respondents’ tendency to regulate their emotions. Respondents answer each item on a 7-point Likert-type scale ranging from 1 (*strongly disagree*) to 7 (*strongly agree*) [60]. Cronbach’s alpha ranged from 0.82 to 0.93 for cognitive reappraisal and from 0.76 to 0.88 for expressive suppression.

#### Hope-related goal processes

The Hope Scale is a 12-item self-report measure of hope measuring two goal-related processes: Agency and Pathways [61]. The Agency subscale assesses the perceived determination to successfully reach one’s goals (e.g., “Even when others get discouraged, I know I can find a way to solve the problem”), and the Pathways subscale measures the perceived ability to identify and develop routes to goals (e.g., “There are lots of ways around every problem”). Responses range from 1 (*definitely false*) to 4 (*definitely true*). The Hope Scale has demonstrated strong psychometric properties [61, 62]. Cronbach’s alpha ranged from 0.79 to 0.85 for agency and from 0.70 to 0.82 for pathways.

#### Cultural processes

Simpatía was measured with the Simpatía Scale [53], an 18-item questionnaire that consists of two factors: simpatía-related positivity/warmth and simpatía-related negativity/conflict avoidance. The Simpatía Scale has demonstrated strong psychometric properties in Latino samples [53]. Cronbach’s alpha was 0.82 for simpatía-related positivity/warmth and 0.83 for simpatía-related negativity/conflict avoidance.

Acculturative stress was measured with the short version of the Padilla Social, Attitudinal, Familial and Environmental (SAFE) Acculturative Stress Measure [55]. The SAFE is composed of 24 items that measure stress as a result of the acculturation process in four contexts: social, attitudinal, familial, and environmental acculturative stress [55]. Responses can include 0 (*not experiences/does not apply*) or ratings of events that may have caused acculturative stress from 1 (*not at all stressful*) to 5 (*extremely stressful*). Cronbach’s alpha was 0.88.

#### Demographic and clinical factors

Demographic and clinical data, including testicular cancer–related treatment information, were assessed via medical record review and self-report. In addition, medical comorbidities and physical health symptoms were recorded; comorbidities were assessed by the Charlson Comorbidity Index (CCI) [63]. The CCI results in a weighted score in which a score of zero indicates no present comorbidities, and a higher score is indicative of more medical comorbidities.

### Data analysis

Sample size determination balanced realistic recruitment estimates and sample requirements for planned analyses and recommendations for pilot research [64–66]. Our target sample size was 50 young adult testicular cancer survivors.

Descriptive statistics were computed to report participant characteristics and summarize indicators of study acceptability, tolerability, and ratings of therapeutic alliance. Time since chemotherapy, demographic variables, and medical comorbidities were considered as potential covariates. In accordance with intention-to-treat principles, multiple imputation was used to impute missing values within SPSS using the automatic method selection function.

Repeated measures ANOVA was used to identify patterns of change in anxiety and depression symptoms as well as psychological processes. Finally, multiple linear regression was used to examine the impact of cultural processes on intervention changes in anxiety and depression symptoms. Anxiety and depression symptoms (post-intervention and 3 months post-intervention) were separately regressed on cultural process variables, controlling for baseline symptoms to account for change over time.

## Results

### Sample characteristics

**Fig. 1:**
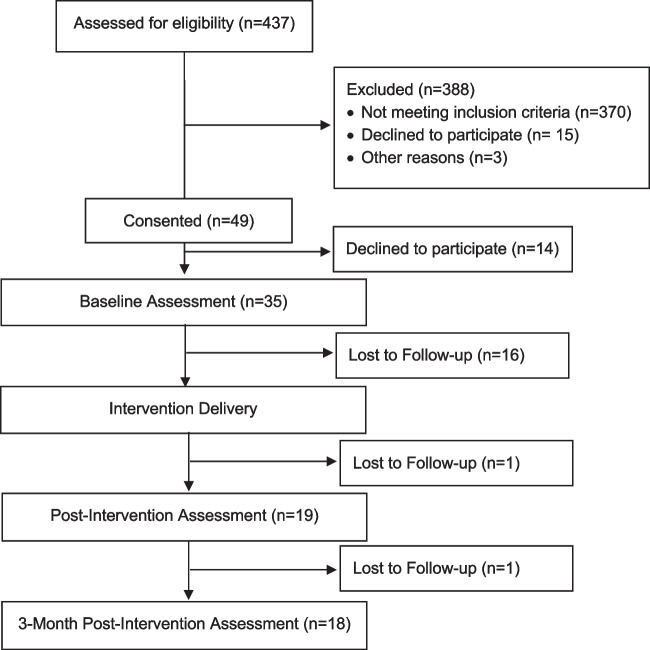
Consort flowchart of participant disposition throughout the study

As depicted in Fig. 1, 35 young adult men completed assessments at baseline. Table 1 outlines the baseline characteristics of the study participants. The mean age of the sample was 29.5 years (SD = 5.54). The majority were of Mexican ethnicity (83%), 45% had a 4-year college degree (or higher), 14% were currently in school, and nearly half (49%) were employed full-time.

**Table 1:** Demographics (*N* = 35)

Age, years (M, SD; range)	29.5, 5.54; 21–38
Ethnic background	
Mexican	83%
Salvadoran	6%
Puerto Rican	3%
Argentinian	3%
Columbian	3%
Cuban	3%
Costa Rican	3%
Ecuadorian	3%
Other	3%
Education	
High school/GED	6%
Vocational training	3%
Some college	31%
2-year college degree	15%
4-year college degree	30%
Graduate	15%
Current student	14%
Household income	
$15,000 or less	13%
$15,001–$30,000	13%
$30,001–$45,000	19%
$45,001–$60,000	16%
$60,001–$75,000	0%
$75,001–$100,000	16%
$100,001 or more	25%
Sexual orientation	
Straight	88%
Gay or bisexual	9%
Other	3%
Relationship status	
Single	49%
Married	36%
Committed/partnered	15%
Have at least 1 child	26%
Lives with parents	40%
Employment	
Employed full-time	49%
Employed part-time	26%
Medical leave/disability	6%
Unemployed	19%
Time since diagnosis (*M* months, SD)	36 (11.59)
Time since chemotherapy (*M* months, SD)	30 (13.33)
Cancer stage	
Stage I	22%
Stage II	44%
Stage III	34%

The average time from completion of chemotherapy to study entry was 30 months (SD = 13.33). Also, all participants had undergone surgical intervention. Few participants (8.5%) reported any medical comorbidities on the CCI, with 97% reporting one or zero co-morbid diagnoses. Therefore, CCI was not statistically controlled.

Mean values of outcome variables and psychological processes are reported in Table 2. At baseline, average anxiety symptoms were above the moderately high/borderline range (*M* = 10.91, SD = 4.96) with 60% reporting anxiety symptoms at levels with possible clinical significance. Depressive symptoms were, on average, in the normal range (*M* = 6.43, SD = 3.84); however, nearly 26% reported symptoms in the moderately high/borderline range, and 14% reported depressive symptoms in the range of possible clinical significance.

**Table 2:** Means and standard deviations for outcome variables and psychological processes at pre-intervention, post-intervention, and 3-month follow-up (*N* = 35)

	*M*	SD	Possible range
Anxiety			0 to 21
Baseline	10.91	4.96	
Post-intervention	8.06	4.14	
3-month post-intervention	7.99	4.82	
Depression			0 to 21
Baseline	6.43	3.48	
Post-intervention	3.03	2.54	
3-month post-intervention	3.92	3.21	
Goal navigation			0 to 2
Baseline	1.36	.44	
Post-intervention	1.78	.27	
3-month post-intervention	1.70	.41	
Goal blockage			0 to 2
Baseline	.78	.52	
Post-intervention	.39	.45	
3-month post-intervention	.35	.37	
Agency			1 to 4
Baseline	2.96	.63	
Post-intervention	3.35	.62	
3-month post-intervention	3.06	.68	
Pathways			1 to 4
Baseline	3.18	.45	
Post-intervention	3.61	.47	
3-month post-intervention	3.57	.46	
Cognitive reappraisal			1 to 7
Baseline	4.92	1.19	
Post-intervention	5.93	1.10	
3-month post-intervention	5.12	1.39	
Expressive suppression			1 to 7
Baseline	3.86	1.40	
Post-intervention	3.23	1.80	
3-month post-intervention	3.08	1.56	

### Acceptability, tolerability, and therapeutic alliance

Initially, 73.1% of eligible men (*n* = 49) consented to participate. Of those eligible men, 52.2% (*n* = 35) completed baseline assessments. Notably, 28.5% of those providing consent completed all study sessions, with the majority of participant loss occurring prior to intervention sessions. Non-initiation of intervention sessions was not significantly correlated with age (*r* =  − 0.23, *p* > 0.05), having college degree (*r* =  − 0.17, *p* > 0.05), household income (*r* = 0.02, *p* > 0.05), being employed (*r* = 0.13, *p* > 0.05), or time since chemotherapy (*r* = 0.13, *p* > 0.05) including baseline levels of depressive (*r* = 0.15, *p* > 0.05) or anxiety (*r* = 0.04, *p* > 0.05) symptoms. Although only 51.4% of those assessed at baseline completed all study procedures, 94.7% of those receiving the intervention completed the remaining study assessments.

As shown in Table 3, participants rated the helpfulness of the intervention skills, as well as the number of and length of intervention sessions, in the moderate to high range. Homework exercises were rated in the moderate range of helpfulness. Participants rated therapist interactions in the high helpfulness range and were very likely to recommend the intervention to a friend with testicular cancer. Finally, therapeutic alliance scores (*M* = 6.17, SD = 0.87; possible range = 0–7) suggested strong rapport and a relatively robust working alliance were established.

**Table 3:** Intervention ratings (*N* = 35)

	*M* (SD)
Helpfulness ratings	
Intervention skills	4.47 (.77)
Number of sessions	4.26 (.81)
Length of sessions	4.33 (.91)
Homework	3.89 (.96)
Therapist interactions	4.79 (.54)
Recommendation	
Likelihood to recommend intervention	4.68 (.58)

Note: the possible range on all items was 0 to 5

### Change in primary outcomes

Examination of change in anxiety symptoms over time revealed a significant effect for time, *F*(2,33) = 5.67, *p* = 0.008, partial *η*^2^ = 0.26, and observed power = 0.83. Pairwise comparisons revealed significant time effects from pre-to-post, post-to-follow-up, and pre-to-follow-up assessments. A similar pattern of change was observed for depressive symptoms. The repeated measures ANOVA revealed a significant effect for time, *F*(2,33) = 14.95, *p* < 0.001, partial *η*^2^ = 0.30, and power = 1.00. Pairwise comparisons revealed significant time effects from pre-to-post, post-to-follow-up, and pre-to-follow-up assessments.

This pattern of results suggests that a significant reduction in both anxiety and depressive symptoms was achieved during the intervention and was maintained three months later (see Table 4).

**Table 4:** Mean difference, standard error, *p-*value, and confidence intervals of the pairwise comparisons from pre-to-post, post-to-follow-up, and pre-to-follow-up assessments on outcome variables and psychological processes using repeated measures ANOVA (*N* = 35)

	Mean difference	Standard error	*p*	95% CI (lower; upper)
Distress outcomes				
HADS-Anxiety				
Pre to post	− 2.85	.84	.002	− 4.59; − 1.15
Post to follow-up	− .07	.45	.878	− .98; .84
Pre to follow-up	− 2.92	.93	.003	− 4.80; − 1.04
HADS-Depression				
Pre to post	− 3.40	.61	< .001	− 4.64; − 2.15
Post to follow-up	.89	.48	.071	− .08; 1.86
Pre to follow-up	− 2.51	.63	< .001	− 3.78; − 1.24
Psychological processes				
Goal navigation				
Pre to post	.42	.08	< .001	.27; .58
Post to follow-up	− .07	.05	.144	− .18; .03
Pre to follow-up	.35	.08	< .001	.19; .50
Goal blockage				
Pre to post	− .39	.11	< .001	− 60; − .17
Post to follow-up	− .04	.05	.465	− .14; .07
Pre to follow-up	− .42	.10	< .001	− .63; − .22
Agency				
Pre to post	.39	.10	< .001	.20; .59
Post to follow-up	− .29	.08	< .001	− .45; − .13
Pre to follow-up	.10	.09	.277	− .09; .30
Pathways				
Pre to post	.43	.10	< .001	.24; .63
Post to follow-up	− .04	.05	.364	− .14; .05
Pre to follow-up	.39	.10	< .001	.18; .60
Cognitive reappraisal				
Pre to post	1.01	.27	< .001	.46; 1.57
Post to follow-up	− .81	.24	.002	− 1.29; .33
Pre to follow-up	.20	.25	.428	− .31; .72
Expressive suppression				
Pre to post	− 6.47	.34	.067	− 1.34; .05
Post to follow-up	− .14	.39	.723	− .93; .65
Pre to follow-up	− .79	.29	.009	− 1.37; − 21

### Psychological processes

Changes in psychological processes—namely, goal navigation (i.e., goal navigation capacity, goal blockage), hope (i.e., agency, pathway mapping), and emotion regulation (i.e., cognitive reappraisal, expressive suppression)—were examined.

Regarding goal navigation processes, goal navigation capacity [*F*(2,33) = 14.69, *p* < 0.001, partial *η*^2^ = 0.47, power = 1.00] and goal blockage [*F*(2,33) = 8.39, *p* = 0.001, partial *η*^2^ = 0.34, power = 0.95] both showed improvements post-intervention that were maintained 3 months post-intervention. Both processes of hope increased with intervention [agency: *F*(2,33) = 10.28, *p* < 0.001, partial *η*^2^ = 0.38, power = 0.98; pathway mapping: *F*(2,33) = 10.41, *p* < 0.001, partial *η*^2^ = 0.39, power = 0.98]. However, these gains were somewhat attenuated in the 3-month follow-up period (see Table 4). Finally, both emotion regulation methods improved with intervention (i.e., greater use of cognitive reappraisal and lessened use of expressive suppressive) [cognitive reappraisal: *F*(2,33) = 8.26, *p* = 0.001, partial *η*^2^ = 0.33, power = 0.95; expressive suppression: *F*(2,33) = 8.26, *p* = 0.019, partial *η*^2^ = 0.21, power = 0.72].

### Cultural factors

The final set of analyses explored the potential that intervention-driven changes in anxiety and depressive symptoms could be conditioned by aspects of simpatía (i.e., positivity/warmth and negativity/conflict avoidance) or acculturative stress. Results are reported in Table 5.

**Table 5:** Cultural predictors of changes in anxiety and depression symptoms (*N* = 35)

	Post-intervention anxiety symptoms			3-month post-intervention anxiety symptoms			Post-intervention depressive symptoms			3-month post-intervention depressive symptoms		
	*B*	SE	β	*B*	SE	β	*B*	SE	β	*B*	SE	β
Simpatía-related positivity and warmth	− 1.30	1.77	− .12	− 2.54	2.07	− .20	− 2.50	1.01	− .38*	− 2.05	1.31	− .24
	*F*(2, 34) = 3.57*; *R*^2^ = .19			*F*(2, 34) = 3.47*; *R*^2^ = .18			*F*(2, 34) = 6.96**; *R*^2^ = .30			*F*(2, 34) = 5.88**; *R*^2^ = .27		
Simpatía-related negativity and conflict avoidance	1.04	1.09	.15	.05	1.31	.01	− .03	.69	− .01	− .48	.84	− .09
	*F*(2, 34) = 3.90*; *R*^*2*^ = .20			*F*(2, 34) = 2.60^†^; *R*^*2*^ = .14			*F*(2, 34) = 3.28*; *R*^*2*^ = .17			*F*(2, 34) = 4.54*; *R*^*2*^ = .22		
Acculturative Stress	1.97	.88	.34*	2.26	1.05	.34*	.97	.56	.27^†^	1.58	.67	.35*
	*F*(2, 34) = 6.35**; *R*^2^ = .28			*F*(2, 34) = 5.29**; *R*^2^ = .20			*F*(2, 34) = 5.09*; *R*^*2*^ = .24			*F*(2, 34) = 7.92**; *R*^2^ = .33		

Note. All analyses controlled for baseline values of anxiety or depressive symptoms

^*^p < .05; **p < .01; ***p < .001

^†^*p* < .10

Simpatía-related positivity and warmth were associated with reductions in depressive symptoms at post-intervention, but not at 3 months. However, relationships with anxiety symptoms were not significant. Simpatía-related negativity and conflict avoidance were not related to changes in distress symptoms. Finally, acculturative stress was significantly associated with greater anxiety at both time points, as well as greater depressive symptoms at the follow-up assessment. Relationships with increased depressive symptoms post-intervention approached significance.

### Post hoc analyses

To provide insight into how cultural processes might be acting on the psychological processes that underscore GET, simpatía-related positivity and warmth and acculturative stress were further tested as predictors of changes in psychological processes (baseline to post-intervention) using multiple linear regression. Reporting only significant associations, simpatía-related positivity and warmth were related to less goal blockage ($\widehat{\beta }$ = − 0.50, *p* = 0.002), less use of expressive suppression ($\widehat{\beta }$ = − 0.48, p = 0.005), and greater skill in pathway mapping ($\widehat{\beta }$ = 0.38, *p* = 0.026). Acculturative stress was associated with diminished goal navigation capacity ($\widehat{\beta }$ = − 0.43, *p* = 0.013), greater goal blockage (β = 0.60, *p* < 0.001), and lower skill in pathway mapping ($\widehat{\beta }$ = − 0.50, *p* = 0.003).

## Discussion

Overall, few behavioral interventions exist to meet the specific needs of young adult cancer survivors, and even fewer have been tailored with a cultural lens [67, 68]. This pilot trial of GET provides strong support for the feasibility and acceptability of this approach to addressing distress in young adult Latino men after testicular cancer, as well as the potential for GET to lead to improvements in goal and emotion regulation skills in this population. There was evidence that participants perceived GET skills, sessions, and interventionists to be moderately or very helpful to them. Likewise, therapeutic alliance ratings were high. In fact, helpfulness and alliance scores surpassed those observed in the general trial [see 49]. We believe this pattern of results is particularly notable in a young adult Latino patient group who historically underutilizes supportive care options.

GET was well tolerated, as nearly all participants who began intervention sessions completed all remaining procedures. However, a sizeable number of participants were lost after completing baseline assessment and before session initiation (46%). This rate is quite high compared to participants in a pilot trial of GET [49]. There are few clear indicators to fully explain this level of drop-out after baseline. Mostly, participants cited time constraints as their primary concern. We did not detect any differences in demographic factors or levels of distress between participants who initiated intervention sessions compared to those who did not. However, in post-study debriefing with study completers, they tended to describe a general desire to participate in a study specific to young Latino men. It may be that study recruitment attracted some survivors with an initial desire to contribute without a personal motivation for intervention.

Because GET is designed for young adults and is focused on the utility of goals, values, and emotions and involves goal navigation in a manner consistent with personal values, GET may feel more consonant with the experiences of Latino survivors. The utilization of trained, young adult, Latino interventionists could also contribute to these ratings and rates of retention. In future studies, efforts will be needed to understand the specific factors that contribute to attrition at various time points.

This trial provides preliminary evidence for GET in addressing psychological distress in young adult Latino male cancer survivors. GET evidenced notable reductions in anxiety and depression (i.e., medium effect sizes) during the course of the intervention that were larger than those observed in the general pilot trial of GET [49]. Moreover, these reductions appear to be maintained in the 3-month follow-up period. Results show a similar pattern across most of the psychological processes thought to drive positive change in GET, with some exceptions. For instance, although the agency increased from pre- to post-intervention, gains were largely lost in the 3-month follow-up period. These results in the context of a single-arm design provide preliminary evidence of GET as a potentially efficacious intervention for young adult Latinos with testicular cancer. Further, they suggest that GET works by way of similar psychological processes as seen in other preliminary studies of GET.

Results suggest that one dimension of simpatía, positivity and warmth, likely influences GET and GET-relevant processes in beneficial ways. Men who scored higher on this dimension of simpatía had greater post-intervention reductions in depressive symptoms, less goal blockage, less use of expressive suppression, and a great ability to map goal pathways. This is the first study to examine these processes in the context of an intervention in this group. Future studies might further consider the role of cultural values to optimize this impact on some individuals. It may be that simpatía-related positivity and warmth facilitates familial and community support and involvement in care and recovery, which in turn mobilizes goal-related resources, enhances coping effectiveness, reduces stigma, and potentially promotes physical and psychological resilience. It is also possible that simpatía-related positivity and warmth is related to motivation toward intervention or a greater likelihood to generally benefit from supportive care intervention. Future studies might also consider if congruence in the level of simpatía between the interventionist and participant influences treatment response.

Acculturative stress emerged as a risk factor for poorer outcomes of GET. Relatively high levels of acculturative stress were associated with increases in anxiety and depressive symptoms after GET. Understanding and addressing how acculturative stress impacts emotional functioning in survivorship will be an important target for future research. It is possible that acculturative stress acts directly on distress outcomes by way of changes in health and coping behaviors, barriers to care and support services, cumulative psychological toll, or disruption in social support networks. It may also be the case that higher acculturative stress is experienced by individuals with fewer socioeconomic resources. These possibilities themselves could impede goal pursuits as well as perceptions of autonomy and agency. It will be important to understand how acculturative stress interacts with the GET approach. In this study, it was associated with diminished goal navigation capacity, greater goal blockage, and lower skill in pathway mapping.

The clarification of one’s values and the identification and pursuit of goals can themselves be culturally laden. This study identified simpatía and acculturative stress as relevant influences. However, it is worth noting that a wide range of values have been of focus in the supportive cancer care literature such as familism, machismo, respeto, and marianismo [see 2]. Formative research underscoring the development of GET identified a preference for positivity, the avoidance of familial conflict, and interpersonal harmony among young adults with testicular cancer, which was pronounced among Latinos [69] and mapped well onto conceptualizations of simpatía. Also underscoring the current study, Latino participants across pilot and feasibility studies described the influence of stressors that map well onto notions of acculturative stress on goal pursuits and perceptions of personal agency. Future research should measure additional cultural influences and values as potential conditioning intervention effects, and we hope our study encourages the inclusion of simpatía and acculturative stress in that consideration.

### Limitations

Results must be viewed in light of several limitations. The primary limitation of this study is its sample size and noncontrolled design. Also, although the study observed significant changes in depressive symptoms, baseline levels of symptoms were in the normal range. Whether reductions in depressive symptoms are clinically meaningful cannot be fully concluded. Notably, the final number of individuals providing consent (*n* = 49) fell short of the target of fifty due to an unexpected declination to participate by the final participant. Finally, patterns of attrition must be considered.

Although all participants were given the option to select the use of English or Spanish in sessions, very few participants chose Spanish. For that reason, more research will be needed to better understand the impact of GET when delivered in Spanish and the influence of simpatía and/or acculturative stress when GET is delivered in Spanish. However, we found that during sessions conducted in English many of our participants in the course of the therapy had moments in which they used Spanish to express a thought or idea. Having a therapist who can switch between English and Spanish appears to have clinical utility.

### Future directions

Further research is needed to establish the efficacy of GET in young adult Latino survivors using a randomized-controlled design with the utilization of a comparison group to either standard care or a time and attention-supportive intervention. Future studies should consider the use of a longer follow-up period, a larger sample size, and the inclusion of young adult Latino survivors across cancer types. In addition to the rich assessment of psychological processes, biological and physiological mechanisms underlying the changes resulting from GET should also be tested [see 46]. Such studies should also systematically examine the contribution of factors affecting study enrollment and retention. Additionally, once the efficacy of GET has been established in the context of a RCT, an important future direction will be to evaluate the delivery of GET via digital modalities.

It should be noted that this pilot study does not reflect a complete cultural adaptation of GET in the sense of focusing on a rigorous tailoring of the intervention. Rather, consistent with the suggestion of models of cultural adaptation of intervention [70–72], this study engaged in information gathering as the first stage in cultural adaptation. In this framework, the current study provides information to understand if/how GET can change outcomes in young adult Latino men after testicular cancer, as well as some insight into culturally relevant influences. Future stages should test a preliminary adapted version of GET built with integrated input from key stakeholders and community partners and engage in iterative processes of intervention refinement. Current results suggest that the initial adaptation of GET might focus on navigating acculturative stress in the pursuit of goals and, to some extent, emphasizing the beneficial aspects of simpatía-related positivity and warmth as an emotion-regulating coping tool.

### Conclusions

Overall, these findings underscore the importance of culturally sensitive and holistic approaches to survivorship interventions that take into account the unique experiences and needs of young adult Latino cancer survivors. By addressing cultural factors and acculturative stress, interventions can be better tailored to promote psychological well-being and resilience in this population. Importantly, existing interventions, including GET, may well be good fits within this cultural context.

## Data Availability

No datasets were generated or analysed during the current study.
